# Dramatic response to pembrolizumab after pseudoprogression in a patient with advanced metastatic castration‐resistant prostate cancer

**DOI:** 10.1002/iju5.12508

**Published:** 2022-08-02

**Authors:** Takumi Kageyama, Norihito Soga, Sho Sekito, Seiichi Kato, Yuji Ogura, Takahiro Kojima, Masahiro Kanai, Takahiro Inoue

**Affiliations:** ^1^ Department of Nephro‐Urologic Surgery and Andrology Mie University Graduate School of Medicine Tsu Mie Japan; ^2^ Department of Urology Aichi Cancer Center Hospital Nagoya Aichi Japan; ^3^ Department of Urology Suzuka General Hospital Suzuka Mie Japan; ^4^ Department of Pathology and Molecular Diagnostics Aichi Cancer Center Hospital Nagoya Aichi Japan

**Keywords:** microsatellite instability, pembrolizumab, prostate cancer, pseudoprogression

## Abstract

**Introduction:**

Prostate cancer with a microsatellite instability‐high or mismatch repair‐deficient status is not common. Few reports of the response to pembrolizumab in metastatic castration‐resistant prostate cancer in a real‐world setting have been reported. This case report describes a dramatic response to pembrolizumab after initial pseudoprogression in a patient with microsatellite instability‐high metastatic castration‐resistant prostate cancer.

**Case presentation:**

A 70‐year‐old man was administered pembrolizumab for metastatic castration‐resistant prostate cancer after the genetic evaluation of lymphadenectomy revealed a microsatellite instability‐high status. His general condition dramatically improved after pseudoprogression. His favorable condition has been maintained for 1 year since the final dose.

**Conclusion:**

We experienced a case of dramatic response to pembrolizumab after pseudoprogression in a patient with advanced metastatic castration‐resistant prostate cancer. In patients with metastatic castration‐resistant prostate cancer and the microsatellite instability‐high/mismatch repair‐deficient phenotype, a few months follow‐up is necessary to evaluate the efficacy of pembrolizumab.

**Abbreviations & Acronyms**
ABI: abiraterone
dMMR: mismatch repair deficiency
ENZ: enzalutamide
mCRPC: metastatic castration‐resistant prostate cancer
MSI‐H: microsatellite instability‐high
PBLZ: pembrolizumab
PD‐1: programmed cell death 1
PSA: prostate‐specific antigen

**Keynote message**

We experienced the first case of a dramatic response to pembrolizumab after initial pseudoprogression in a patient with microsatellite instability‐high metastatic castration‐resistant prostate cancer. A few months follow‐up is necessary to evaluate the efficacy of pembrolizumab.

## Introduction

PBLZ is approved for treating advanced chemotherapy‐refractory malignancies with an MSI‐H or dMMR status. The objective response rates of PBLZ in mCRPC are 3–5% regardless of programmed cell death ligand 1 expression.^1^ Although the response rates to anti‐PD‐1 therapy are low in unselected patients with mCRPC, patients with an MSI‐H or dMMR status are more likely to have favorable responses.^2^ However, because few patients with mCRPC have MSI‐H/dMMR, few reports of the response to PBLZ in patients with mCRPC in a real‐world setting are available. We present a case of a dramatic response to PBLZ in a patient with MSI‐H mCRPC.

## Case presentation

A 70‐year‐old man was first diagnosed in 2009 with Gleason 4 + 4 = 8 metastatic prostate cancer and bone metastases in the left second rib. His PSA level was 305 ng/mL. Combined androgen blockade was administered. He was diagnosed with mCRPC after 1 year, and additional sequential hormonal therapy was provided.

**Fig. 1:**
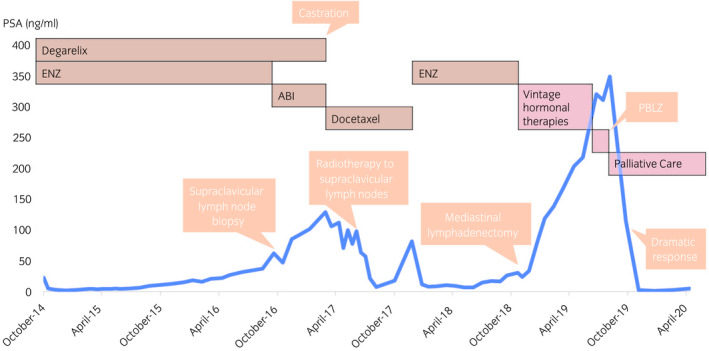
PSA levels across the patient's treatment course after novel hormonal therapy administration. Vintage hormonal therapies included bicalutamide, estramustine, and ethinylestradiol.

**Fig. 2:**
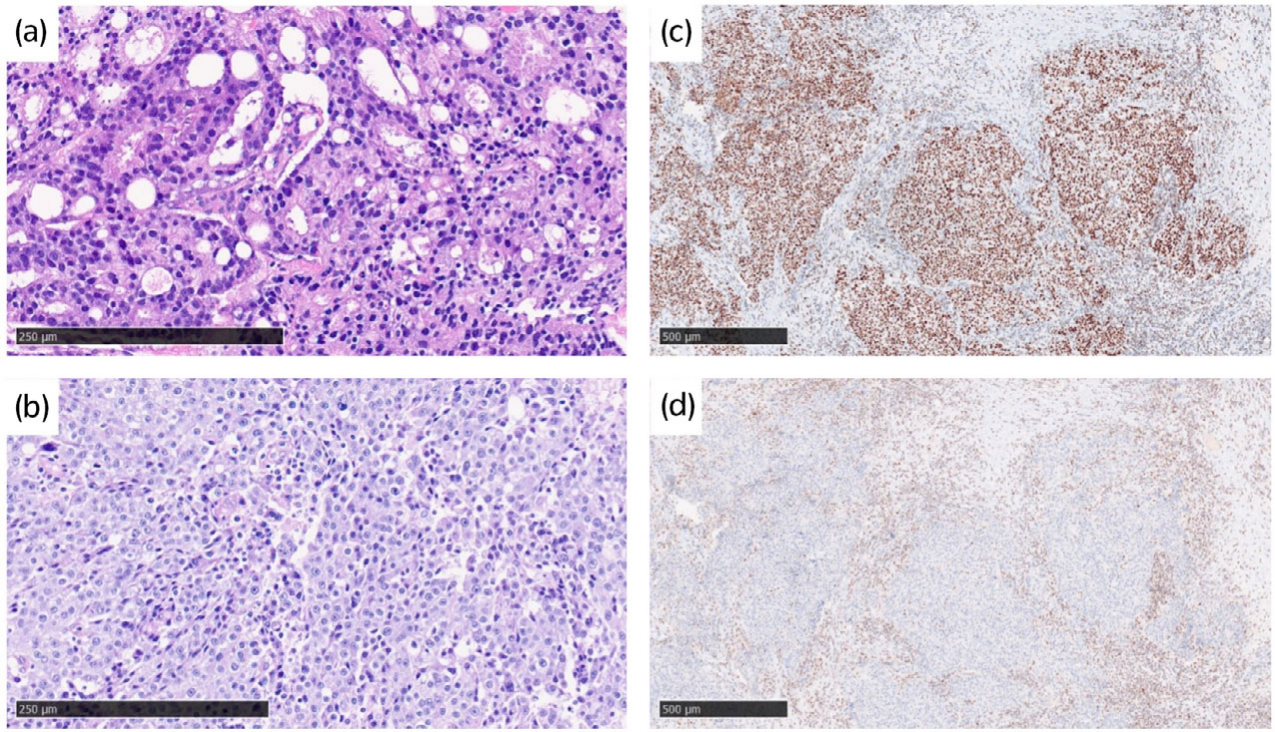
(a) Pathological findings of left supraclavicular lymphadenopathy. In the histopathological examination, hematoxylin and eosin staining revealed the proliferation of atypical cells with a high nucleo‐cytoplasmic ratio and cribriform pattern. (b–d) Pathological findings of anterior mediastinal lymphadenopathy. Histopathological examination revealed a poorly differentiated carcinoma, differing from the prostate cancer histology observed in previous lymph node biopsy specimens (b). Immunohistochemical staining demonstrated that tumor cells were positive for NKX 3.1 and PSMA but negative for TTF‐1, PSX8, p40, CD5, and PSA, leading to a diagnosis of metastasis of prostate cancer. Furthermore, they were positive for MSH6 (c) but had a low expression for PMS2 (d), indicating a mismatch repair gene abnormality.

The clinical course after novel hormonal therapy administration is presented in Figure 1. ENZ 160 mg/day plus degarelix was started in 2014, and the patient's PSA level decreased, permitting disease control for 2 years. However, PSA progression and metastatic left supraclavicular lymphadenopathy occurred, as pathologically diagnosed by needle biopsy (Fig. 2a). ABI 1000 mg/day plus prednisone 10 mg/day was ineffective, and he underwent surgical castration and docetaxel therapy. Because of left supraclavicular lymph node enlargement, he received palliative radiotherapy (30 Gy/10 Fr). The chemoradiotherapy decreased his PSA level and the size of the lymph node for a few months.

Although the ENZ re‐challenge suppressed the patient's PSA level for 6 months, anterior mediastinal lymphadenopathy appeared. Because we could not exclude the possibility of other malignancies as the cause of extra‐regional lymphadenopathy, lymphadenectomy was performed, and the patient was diagnosed with poorly differentiated cancer from the original prostate cancer (Fig. 2b–d).

**Fig. 3:**
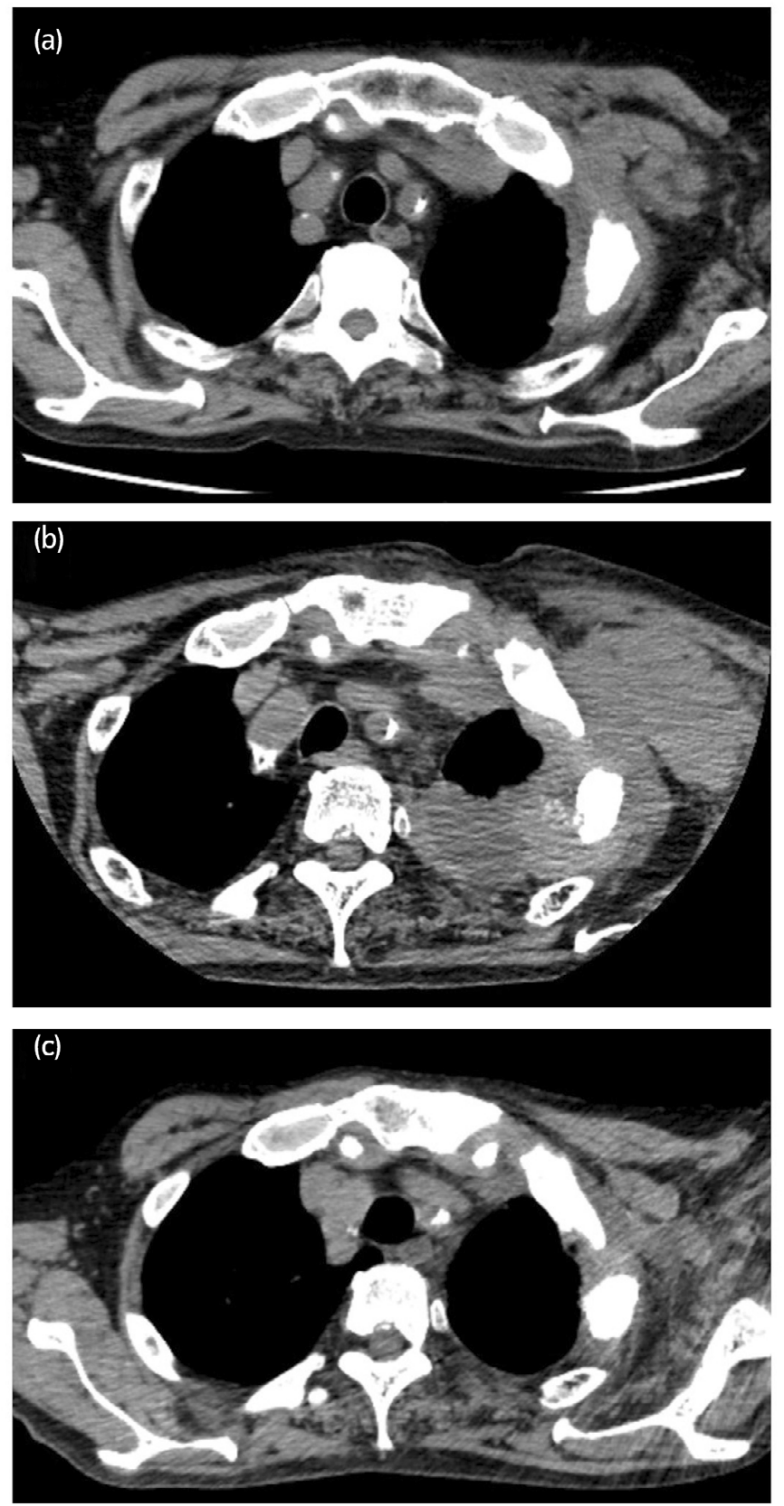
Tumor response to pembrolizumab. (a) Computed tomography showed a swelling of the left axillary lymph node, measuring 46.6 × 23.3 mm, before PBLZ administration. (b) After four cycles of PBLZ, the left axillary lymph nodes were severely enlarged, measuring 74.6 × 39.5 mm (an increase of 160% from baseline tumor burden), and malignant left pleural effusion was observed. (c) Five months after the final dose, the left lymph node adenopathy, and the pleural effusion were dramatically diminished.

Genetic evaluation of the mediastinal lymphadenectomy specimen permitted a diagnosis of MSI‐H; thus, the PD‐1 checkpoint inhibitor PBLZ was adapted for this case. However, after four cycles of PBLZ 200 mg intravenously every 3 weeks, further enlargement of the left axillary lymph nodes was detected (Fig. 3a,b). Consequently, his general condition worsened, and severe pain caused to the left upper extremity edema occurred. Palliative treatment with oxycodone 40 mg/day was started. In addition, oral hydrocortisone was needed for Grade 2 adrenocortical insufficiency attributable to an immune‐related adverse event.

Because of the patient's drastically worse performance status, no further treatment was adopted, and he consented to receive the best supportive care. However, 2 months after the end of PD‐1 treatment, the left upper extremity edema and severe pain had dramatically improved. Furthermore, axillary lymph node enlargement and other metastases had diminished (Fig. 3c), in addition to a drastic decrease of his PSA level after 5 months. His general condition has remained improved for 1 year since the final dose of PBLZ without additional treatment.

## Discussion

PBLZ was recently approved for use in patients with metastatic MSI‐H/dMMR solid tumors. In patients with advanced dMMR cancers across tumor types, objective radiographic responses to PBLZ were observed in 53% of patients.^3^ In prostate cancer, PBLZ has exerted durable anti‐tumor activity in patients who progressed on endocrine therapies and chemotherapies.^4^ In addition, some dramatic responses to PBLZ have been reported.^5^, ^6^, ^7^, ^8^, ^9^ However, because only 2.7–8.1% of patients with mCRPC have an MSI‐H/dMMR status,^10^, ^11^, ^12^ the general clinical course after PBLZ administration in mCRPC has not been clarified.

In previous reports, 51.4% of patients with MSI‐H/dMMR mCRPC displayed a greater than 50% decline in PSA levels, including 40% patients with >90% declines, and 35% achieved objective radiographic responses.^2^, ^13^, ^14^ Graham *et al*.^2^ and Abida *et al*.^13^ used tumor tissue to observe the prevalence of MSI‐H/dMMR histologically, whereas Barata *et al*.^14^ used liquid biopsy to assess the MSI status with circulating tumor DNA. The detection rate and effectiveness of the two methods were similar. To get a valuable specimen for genetic evaluation, metastasectomy under aggressive operation should be sometime indispensable, like in this case.

PBLZ has recently also been approved for patients with tumor mutational burden high advanced solid tumors. Furthermore, it has also been reported that immune checkpoint inhibitors may be more effective in cyclin‐dependent kinase 12‐altered prostate cancer.^15^ Examining genetic information to select the proper treatment for advanced prostate cancer is essential.

Checkpoint inhibitor therapy produces a unique response pattern termed pseudoprogression, which includes increased tumor lesions with tumor necrosis and a subsequent decrease in tumor volume.^16^ The rate of pseudoprogression across solid tumors ranged 0.6–15.8% in clinical trials.^17^ In previous reports, pseudoprogression was not observed in mCRPC.^18^ The maximum reported increase in the tumor burden associated with pseudoprogression had ranged 20–163%.^19^ Some patients responded later than 12 weeks after the initial increase in the total tumor burden or during or after the appearance of new lesions.^20^ As reported previously, a long interval of 2 months was needed to observe objective and subjective improvement after four doses of PBLZ in this case. In the future, novel criteria for evaluating immunotherapeutic responses should be developed.

## Conclusion

To our knowledge, this is the first case of a dramatic response to PBLZ after initial pseudoprogression in a patient with MSI‐H mCRPC. In patients with mCRPC and the MSI‐H/dMMR phenotype, a few months follow‐up is necessary to evaluate the efficacy of PBLZ.

## Author contributions

Takumi Kageyama: Conceptualization; data curation; formal analysis; writing – original draft. Norihito Soga: Data curation; project administration; supervision; writing – review and editing. Sho Sekito: Data curation. Seiichi Kato: Data curation. Yuji Ogura: Data curation. Takahiro Kojima: Supervision. Masahiro Kanai: Supervision. Takahiro Inoue: Supervision; writing – review and editing.

## Conflict of interest

The authors declare no conflict of interest.

## Approval of the research protocol by an Institutional Reviewer Board

N/A.

## Informed consent

Written informed consent was obtained from the patient for publication of this report.

## Registry and the Registration No. of the study/trial

N/A.
